# Evaluation and comparison of antimicrobial property of chitosan nanoparticles on nickel-titanium archwires: an *in vivo* study

**DOI:** 10.1590/2177-6709.31.1.e2625184.oar

**Published:** 2026-04-17

**Authors:** Jainil PATEL, Padmaja SHARMA, Kalpesh PATEL

**Affiliations:** 1 Manubhai Patel Dental College & Dental Hospital, Department of Orthodontics (Vadodara/Gujarat, India).

**Keywords:** Chitosan nanoparticles, Nanocoating, Orthodontic wires, White spot lesions, Antibacterial properties, Nanopartículas de quitosana, Nanorevestimento, Fios ortodônticos, Lesões de mancha branca, Propriedades antibacterianas

## Abstract

**Introduction:**

Fixed orthodontic appliances can serve as a breeding ground for bacterial accumulation and colonization, which are responsible for the development of white spot lesions (WSLs).

**Objective:**

This study evaluated and compared the antibacterial activity of chitosan nanoparticle-coated and uncoated nickel-titanium (NiTi) orthodontic archwires against *Streptococcus mutans* (*S. mutans*) and *Lactobacillus acidophilus (L. acidophilus)*.

**Material and Methods:**

Thirty patients received 0.016-in NiTi archwires, using a split-mouth design. Sixty archwires were randomized into two groups: Group A, nanochitosan-coated (n = 30, experimental), and Group B, uncoated (n = 30, control). Allocation was determined using sequentially numbered, opaque, sealed envelopes prepared by an independent researcher, with assignments concealed until allocation. The ionic gelation technique was used for coating. After six weeks in the oral cavity, archwires were retrieved, and bacterial presence was evaluated as Colony Forming Units (CFUs), through culture formation.

**Results:**

A statistically significant difference was observed in the mean CFU counts of *S. mutans* and *L. acidophilus* between the experimental and control groups (*p* < 0.001). The mean CFU values for *S. mutans* were 0.12 × 10^6^ CFU/ml (SD ± 0.38; experimental) and 1.82 × 10^6^ CFU/ml (SD ± 0.69; control). For *L. acidophilus*, mean CFUs were 0.036 × 10^6^ CFU/ml (SD ± 0.06; experimental) and 1.96 × 10^6^ CFU/ml (SD ± 0.65; control).

**Conclusion:**

Orthodontic archwires coated with nanochitosan demonstrated significantly greater antimicrobial activity against *S. mutans* and *L. acidophilus* compared with uncoated archwires.

## RESUMO

**Introdução:**

Os aparelhos ortodônticos fixos podem servir como um ambiente propício para o acúmulo e colonização bacteriana, responsáveis pelo desenvolvimento de lesões de mancha branca (LMBs).

**Objetivo:**

Este estudo avaliou e comparou a atividade antibacteriana de fios ortodônticos de níquel-titânio (NiTi), revestidos e não revestidos com nanopartículas de quitosana, contra bactérias *Streptococcus mutans* (*S. mutans*) e *Lactobacillus acidophilus* (*L. acidophilus*).

**Material e Métodos:**

Trinta pacientes receberam arcos ortodônticos de NiTi de 0,016”, utilizando um desenho de boca dividida. Sessenta arcos foram randomizados em dois grupos: Grupo A, revestidos com nanoquitosana (n = 30, experimental) e Grupo B, não revestidos (n = 30, controle). A alocação foi determinada utilizando-se envelopes opacos, selados e numerados sequencialmente, preparados por um pesquisador independente, com as atribuições mantidas em sigilo até o momento da alocação. A técnica de gelificação iônica foi utilizada para o revestimento. Após seis semanas na cavidade bucal, os arcos foram removidos e a presença bacteriana foi avaliada em Unidades Formadoras de Colônias (UFCs), por meio de cultura bacteriana.

**Resultados:**

Observou-se uma diferença estatisticamente significativa na média da contagem de UFC de *S. mutans* e *L. acidophilus* entre os grupos experimental e controle (p< 0,001). Os valores médios de UFC para *S. mutans* foram 0,12 × 10^6^ UFC/ml (DP ± 0,38; experimental) e 1,82 × 10^6^ UFC/ml (DP ± 0,69; controle). Para *L. acidophilus*, a média de UFC foi de 0,036 × 10^6^ UFC/ml (DP ± 0,06; experimental) e 1,96 × 10^6^ UFC/ml (DP ± 0,65; controle).

**Conclusão:**

Os arcos ortodônticos revestidos com nanoquitosana demonstraram atividade antimicrobiana significativamente maior contra *S. mutans* e *L. acidophilus*, em comparação com arcos ortodônticos sem revestimento.

## INTRODUCTION

Fixed orthodontic appliances are widely used to improve oral function and enhance facial aesthetics. However, their placement substantially increases the risk of dental caries, due to the accumulation of dental plaque around components such as brackets and bands.^1^ ^,^ ^2^ Among the bacteria involved, *Streptococcus mutans* and *Lactobacillus* are the most prevalent. These bacteria produce lactic acid, which promotes enamel demineralization and frequently results in white spot lesions (WSLs), visible as unesthetic, opaque areas on the tooth surface.^3^ ^,^ ^4^

Multiple preventive strategies have been investigated to reduce WSL development during orthodontic treatment, including: patient-dependent measures such as oral hygiene education, antibacterial or fluoride mouth rinses, fluoride-based toothpastes, and probiotic supplements.^5^ ^,^ ^6^ Although effective, such methods rely heavily on patient compliance. To address this limitation, non-compliance-dependent interventions have been introduced, such as fluoride varnishes, fluoride-releasing adhesives, and fluoride-containing orthodontic components.^7^

Recently, nanoparticles have been studied for their antibacterial potential in Orthodontics, either when incorporated into adhesives or applied as coatings on brackets and archwires.^8^ ^-^ ^11^ Among these, chitosan, a naturally occurring biopolymer, has attracted considerable interest because of its biodegradability, biocompatibility, and strong antimicrobial activity.^12^ It has been shown to inhibit bacterial growth, reduce plaque formation, and prevent bacterial adhesion, particularly in *S. mutans*.^12^ ^,^ ^13^ In nanoparticle form, chitosan demonstrates enhanced antibacterial efficacy and has been applied in diverse biomedical and technological fields, including drug delivery, artificial organ development, and washable textiles.^12^ ^-^ ^16^ Furthermore, nanochitosan has exhibited immunostimulatory properties when used in DNA vaccine delivery.^17^

Although the antimicrobial effects of nanochitosan are well documented, limited evidence exists regarding its impact on oral pathogens such as *Streptococci* and *Lactobacilli*, which play central roles in dental caries development. Therefore, the aim of this study was to evaluate and compare the antibacterial activity of nickel-titanium (NiTi) orthodontic archwires coated with chitosan nanoparticles against *S. mutans* and *Lactobacillus acidophilus*. 

## MATERIAL AND METHODS

This prospective study was conducted following ethical approval from the Institutional Ethics Committee (IEC) for Research (Approval no. IEC/MPDC_271/ORTHO-58/23). 

### INCLUSION CRITERIA

1. Patients who provided informed consent to participate in the study.
2. Individuals requiring fixed orthodontic treatment.
3. Patients with good oral hygiene and a healthy periodontal condition.

### EXCLUSION CRITERIA

1. Patients who declined to provide informed consent.
2. Individuals with poor oral hygiene.
3. Patients with periodontal disease or compromised periodontal health.
4. Individuals with systemic conditions such as diabetes mellitus or AIDS, or taking medications like phenytoin or immunosuppressants.
5. Patients with missing anterior teeth.
6. Individuals presenting with localized gingival disease.

### ORAL HYGIENE INSTRUCTIONS

Throughout the study, all participants were instructed to maintain standard oral hygiene. Patients were advised to brush twice daily with a toothpaste, using the modified Bass technique. The use of mouth rinses, probiotics, or additional antimicrobial agents was restricted, to prevent potential confounding effects on bacterial counts.

### SAMPLE SIZE CALCULATION

Considering a clinically relevant mean difference of 5.5 in *Streptococcus mutans* CFU counts between the two groups, with an assumed standard deviation of 7, based on previously reported variability, the required sample size was calculated using the formula:

$$\text{n}=\frac{2\times {\left(Z_{\alpha /2}+Z\beta \right)}^{2}\times \sigma^{2}}{d^{2}}$$

At a 95% confidence level (Z_α/2_ = 1.96) and 80% power (Zβ = 0.84), the minimum sample size was estimated to be 26 per group. To account for possible attrition, 30 samples per group (total n = 60) were included.^9^ ^,^ ^10^

### ALLOCATION METHOD

This was a split-mouth *in vivo* study. Thirty patients undergoing orthodontic treatment who had given consent were selected. Each participant was treated with 0.022 x 0.028-in slot metal MBT (McLaughlin-Bennett-Trevisi) bracket system (Ormco, USA).

Randomization of arches to experimental or control groups was carried out using sequentially numbered, opaque, sealed envelopes prepared by a researcher not involved in clinical procedures. To ensure allocation concealment, the envelopes were opened sequentially only at the time of archwire placement. Thus, one arch of each patient received an uncoated 0.016-in NiTi archwire (control group), while the contralateral arch received a nanochitosan-coated 0.016-in NiTi archwire (experimental group).

### SURFACE MODIFICATION

Chitosan particles (molecular weight 3,800-20,000 Da) were obtained from HiMedia Laboratories Pvt. Ltd., Mumbai, India. A chitosan solution was prepared using the ionic gelation method by dispersing the particles in a 0.25% acetic acid solution, followed by overnight magnetic stirring and dilution with distilled water.^18^ The nanochitosan formulation was developed by the White Lab Material Research Centre, Chennai, India.

**Figure 1:**
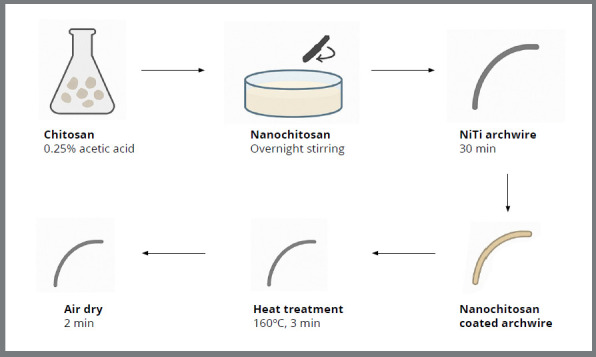
Nanochitosan preparation and surface modification workflow.

Surface modification of the archwires for chitosan nanoparticle coating was performed using the sol-gel technique. Wires were immersed in 100 mL of chitosan nanoparticle solution for 30 minutes, air-dried for 2 minutes, and heat-treated in a hot-air oven at 160°C for 3 minutes. Polyetherimide (PEI) was employed as a binding agent to enhance the adhesion of nanoparticles to the wire surface. Nanoparticle size ranged from 100 to 200 nm (Fig. 1).^19^

### SAMPLE COLLECTION

Patients received 0.016-in NiTi archwires (coated and uncoated) for six weeks. After six weeks, archwires were retrieved, cut at the midline, and stored in saline-filled tubes, for microbiological testing (Genexis Biotech Pvt. Ltd., Vadodara, India).

**Figure 2:**
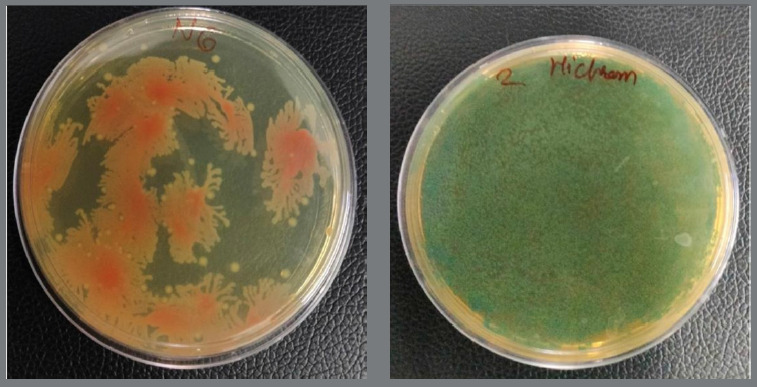
Chrome agar plates showing bacterial growth.

**Figure 3:**
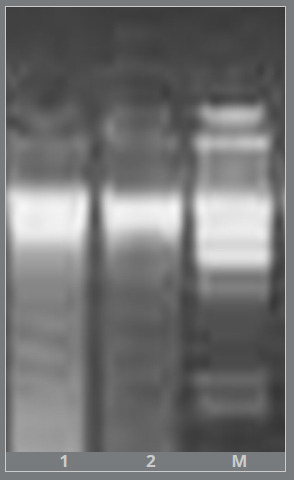
Genomic DNA extraction.

Samples were inoculated in Luria broth for the isolation of *Streptococcus mutans* and *Lactobacillus acidophilus*. Cultures were serially diluted and plated onto chrome agar for bacterial identification (Fig. 2). Colonies of different colours were further inoculated in Luria broth and incubated overnight at 37°C. The bacterial isolates were then used for genomic DNA (gDNA) extraction. Genomic DNA was isolated using a commercial DNA extraction kit. Amplification of the 16S rRNA gene was performed using universal primers specific for 16S rDNA. PCR products were confirmed by electrophoresis on a 0.8% agarose gel (Fig. 3). 

**Figure 4:**
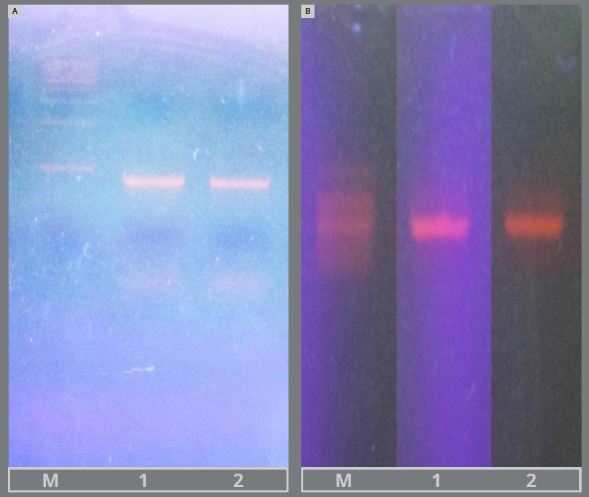
PCR amplification. **A)** *S. mutans*, **B)** *L. acidophilus*.

The amplified DNA was purified using a PCR cleanup kit, and the resulting product was sent for sequencing (Fig. 4). The PCR protocol included: initial denaturation at 94°C for 3 minutes, followed by 35 cycles of denaturation at 94°C for 45 seconds, annealing at 50°C for 60 seconds, and extension at 72°C for 90 seconds. A final extension step was performed at 72°C for 10 minutes, after which the samples were held at 4°C.^20^

Following sequencing and confirmation, antimicrobial activity was assessed by determining bacterial viability through Colony Forming Unit (CFU) counts. Each sample was plated in duplicate, and the mean was recorded. All measurements were performed by a single calibrated observer, to avoid inter-observer variability.

This was a double-blind study: both the technician and participants were blinded to group allocation. Samples sent for microbiological testing were coded, to maintain blinding. 

## STATISTICAL ANALYSIS

Descriptive and inferential statistics were analyzed using the Statistical Package for the Social Sciences (SPSS, version 26.0; IBM Corp., Armonk, NY, USA). A p-value < 0.05 was considered statistically significant. Descriptive statistics for the CFU, including the means and standard deviations (SD), were calculated for each group. 

The Shapiro-Wilk test was used to assess data normality. As the data were not normally distributed (p < 0.05), non-parametric tests were applied. Given the split-mouth design, in which each patient contributed with one arch to the experimental group and the contralateral arch to the control group, paired comparisons were required. Therefore, the Wilcoxon signed-rank test was used to compare CFU counts between the experimental and control arches.

## RESULTS

**Figure 5:**
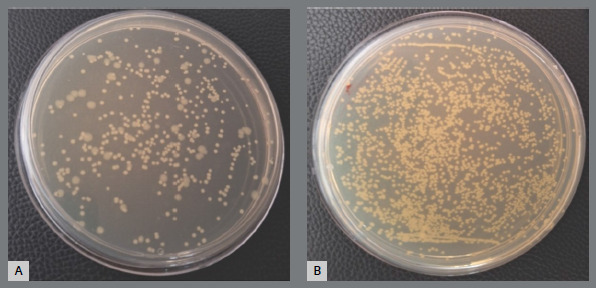
S. mutans colonies: A) Experimental group, B)control group.

Figures 5A and 5B illustrate *Streptococcus mutans* colonies cultured from experimental and control wires, respectively.

**Figure 6:**
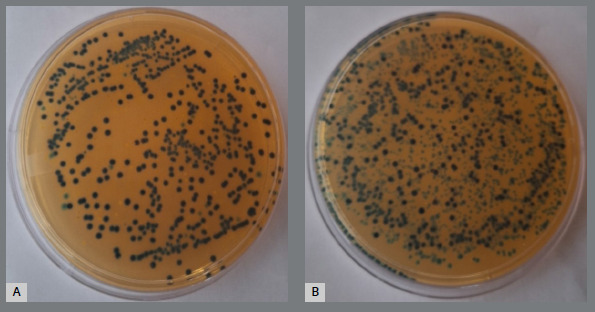
L. acidophilus colonies: A) Experimental group, B) control group.

Figures 6A and 6B illustrate *Lactobacillus acidophilus* colonies cultured from experimental and control wires, respectively.

### STREPTOCOCCUS MUTANS

The mean CFU count in the experimental group (nanochitosan-coated wires) was 0.12 × 10^6^ CFU/ml (SD ± 0.38), compared with 1.82 × 10^6^ CFU/ml (SD ± 0.69) in the control group. This corresponded to a mean reduction of 1.70 × 10^6^ CFU/ml. The Wilcoxon signed-rank test confirmed a highly significant difference between the experimental and control groups (Z = - 4.706, p < 0.001). Out of 30 paired observations, 29 showed lower values in the experimental group, compared to the control, while none showed higher values and 1 was tied. The 95% confidence interval (CI) for the median difference ranged from -1.8 to -1.5 × 10^6^ CFU/ml.

Descriptive statistics and test results are summarized in Tables 1A and 1B. 

**Table 1A:** Descriptive statistics of Colony Forming Units (in 10^6^ CFU/ml) of *S. mutans* of experimental and control groups.

Group	Mean	SD	Q1	Median	Q3	Min	Max	Mode
Control	1.82	0.69	1.1	2.1	2.1	1.0	3.1	2.1
Experimental	0.12	0.38	0.012	0.021	0.098	0.001	2.1	0.021

SD: Standard deviation, Q1: First quartile, Q3: Third quartile.

[Table t1b]

**Table 1B: t1b:** The Wilcoxon Signed Ranks Test for Colony Forming Units of *S. mutans* (Experimental vs. Control Groups).

	n	Mean Rank	Sum of Ranks	Z	P value
Negative Ranks	29^a^	15.00	435.00	-4.706	<0.001
Positive Ranks	0^b^	0.00	0.00		
Ties	1^c^	-	-		

a. Experimental group < Control group. b. Experimental group > Control group. c. Experimental group = Control group.

### LACTOBACILLUS ACIDOPHILUS

For *L. acidophilus*, a similar trend was observed. The mean CFU count in the experimental group was 0.36 × 10^6^ CFU/ml (SD ± 0.06), whereas the control group showed 1.96 × 10^6^ CFU/ml (SD ± 0.65). This corresponds to a mean reduction of 1.60 × 10^6^ CFU/ml. The Wilcoxon signed-rank test again demonstrated a highly statistically significant difference between the experimental and control groups (Z = -4.783, p < 0.001). All 30 paired observations showed lower values in the experimental group, compared to the control, with no higher values and no ties. The 95% confidence interval (CI) for the median difference ranged from -1.84 to -1.36 × 10^6^ CFU/ml.

Detailed descriptive values and intergroup comparisons are provided in Tables 2A and 2B.

**Table 2A:** Descriptive statistics of Colony Forming Units (in 10^6^ CFU/ml) of *L. acidophilus* in control and experimental groups.

Group	Mean	SD	Q1	Median	Q3	Min	Max	Mode
Control	1.96	0.65	1.325	2.1	2.3	1.0	3.2	2.1
Experimental	0.036	0.06	0.0013	0.013	0.022	0.00011	0.22	0.021

SD: Standard deviation, Q1: First quartile, Q3: Third quartile.

[Table t2b]

**Table 2B: t2b:** Wilcoxon Signed Ranks Test for Colony Forming Units of *L. acidophilus* (Experimental vs. Control Groups).

	n	Mean Rank	Sum of Ranks	Z	P value
Negative Ranks	30^a^	15.50	465.00	-4.783	<0.001
Positive Ranks	0^b^	0.00	0.00		
Ties	0^c^	-	-		

a. Experimental group < Control group. b. Experimental group > Control group. c. Experimental group = Control group.

**Figure 7:**
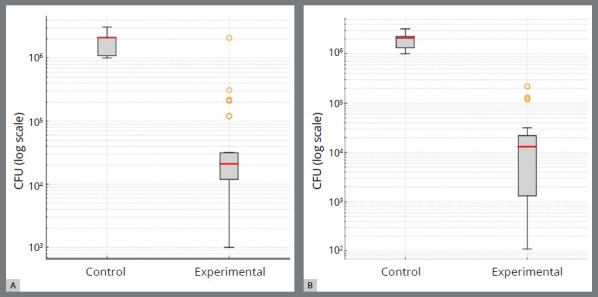
Box plot: **A)** *S. mutans*, **B)** *L. acidophilus*.

Box plot analysis further illustrated the differences in CFU counts between groups. For *S. mutans*, the control group clustered tightly around higher CFU counts (approximately 10^6^CFU/ml), while the experimental group displayed substantially reduced medians (approximately 10^4^ CFU/ml) with a broader spread and multiple outliers (Fig. 7A). Similarly, for *L. acidophilus*, the control archwires showed consistently higher values with a narrow interquartile range around 10^6^ CFU/ml, whereas the experimental archwires demonstrated markedly lower median values (around 10^4^ CFU/ml) with a wider distribution and several outliers (Fig. 7B). 

## DISCUSSION

The primary objective of fixed orthodontic treatment is to align teeth and improve the appearance of the smile. However, a frequent complication is the formation of white spot lesions (WSLs).^2^ ^,^ ^3^ Brackets and other components of fixed appliances create irregular surfaces that tend to accumulate dental plaque. Inadequate oral hygiene allows plaque to persist, leading to enamel demineralization and the subsequent development of WSLs.^2^ ^-^ ^4^

Cariogenic bacteria, particularly *Streptococcus mutans* and lactic acid-producing species, are key contributors to the development of dental caries.^1^ Orthodontic treatment alters the oral environment by lowering pH and creating additional niches that promote the accumulation of *S. mutans*, *Lactobacillus* species, and food debris.^2^ These microbial changes often increase salivary concentrations of cariogenic bacteria, which may explain the enamel decalcification and WSLs observed after orthodontic therapy.^1^ ^-^ ^4^ Therefore, reducing bacterial adherence to orthodontic appliances is essential to minimize the risk of enamel demineralization.

Chitosan, a naturally occurring polymer derived from chitin, is considered safe for biological applications due to its low toxicity and biocompatibility.^21^ Chitosan nanoparticles (CS-NPs) offer several advantages over other nanomaterials, including biodegradability, natural origin, mucoadhesive properties, and ease of chemical modification, which make them highly promising for biomedical and drug delivery applications.^21^ Chitosan is widely recognized for its strong antibacterial properties, and has been effectively utilized in biomedical engineering in numerous forms, including nanoparticles, nanocomposites, colloids, fibers, and even as a food preservative, to inhibit biofilm formation and reduce the incidence of infections.^22^ In addition to its antibacterial activity, nanochitosan also exhibits antifungal and antiviral effects, and does not contribute to the resistance problems commonly associated with certain antibiotics.^22^ ^-^ ^24^

CS-NPs can be synthesized by various techniques, including ionic gelation,^18^ reverse micellar method,^25^ microemulsion,^26^ emulsion droplet coalescence,^27^ and spray drying.^28^ Among these, ionic gelation - first described by Calvo et al.^18^ - is considered the most suitable for biomedical applications. In the present study, CS-NPs were synthesized using the ionic gelation method, while the coating was performed using a sol-gel thin-film dipping technique. This approach provides uniform structure, high purity, and stability for chemically sensitive particles, while allowing control over porosity and conductivity.^15^ ^,^ ^25^ ^-^ ^28^

The antimicrobial activity was assessed using CFU counts for *S. mutans* and *L. acidophilus*. The nanochitosan-coated archwires demonstrated significantly lower bacterial counts than controls, confirming their antibacterial efficacy. These findings are consistent with previous studies demonstrating that incorporation of CS-NPs into dental and orthodontic materials enhances antibacterial properties.^29^ ^-^ ^31^ Sodagar et al.^30^ reported that CS-NPs containing composites exhibited strong antibacterial effects against cariogenic bacteria, without compromising mechanical properties. Similarly, Hosseinpour Nader et al.^31^ confirmed the antibacterial efficacy of orthodontic primers containing chitosan nanoparticles *in vivo*, reporting reduced bacterial colonization in multispecies biofilms. The current study extends this evidence to orthodontic archwires, highlighting that surface modification with chitosan nanoparticles can provide antibacterial benefits directly at the wire-bracket interface, where plaque tends to accumulate most.

The antimicrobial action of chitosan involves multiple mechanisms: it inhibits bacterial enzymes, chelates essential metal ions, and forms polyelectrolyte complexes on the bacterial cell wall.^10^ In this study, *L. acidophilus* showed greater susceptibility than *S. mutans*. This may be due to structural differences: Gram-positive *L. acidophilus* relies on teichoic acids and peptidoglycan, which are more susceptible to disruption by the cationic amino groups of chitosan.^24^ In contrast, *S. mutans* has additional acidogenic and aciduric adaptations that enhance survival in acidic environments. Such variations could explain the differential susceptibility. Similar findings have been reported in earlier studies showing that chitosan reduces plaque regrowth and interferes with bacterial adhesion to dental surfaces.^12^ ^,^ ^13^

From a clinical standpoint, these antibacterial effects may be relevant for reducing risk factors associated with the development of WSLs, a common complication of fixed orthodontic treatment. Elevated salivary levels of *S. mutans* and *Lactobacillus* species have been correlated with WSL development in orthodontic patients.^1^ ^-^ ^4^ By reducing bacterial adhesion and viability on orthodontic archwires, nanochitosan coatings may reduce the microbial challenge at critical plaque-retentive sites, potentially decreasing the incidence of WSLs during treatment.

This study is, to our knowledge, among the first *in vivo* investigations to assess the antimicrobial effects of CS-NPs coatings on NiTi orthodontic archwires. Whereas earlier research focused on adhesives, composites, or impression materials,^29^ ^-^ ^31^ this study expands the application to archwire surface modification, supporting the development of non-compliance-dependent antimicrobial orthodontic components.

## LIMITATIONS

1. Further research with larger sample sizes, longer follow-up, and more extensive toxicity assessments is needed to confirm the safety and efficacy of CS-NPs.
2. Plaque accumulation is influenced by surface roughness, which may affect wire properties and contribute to WSLs. Future studies should investigate coating durability, surface characteristics, and corrosion resistance.
3. Green synthesis methods using plant-derived biomolecules could provide an environmentally friendly alternative for CS-NP production.
4. Leaching of nickel due to reaction with chitosan should be evaluated. The acid contained in different food and beverages can release nickel ions from the wire. Oral manifestations of nickel allergy include stomatitis, common perioral rash, angular cheilitis, burning sensation, gingival hyperplasia, erythema multiforme and loss of taste, among other symptoms.^32^
5. The integrity of the nanochitosan coating after six weeks intraoral exposure was not directly verified. Future research employing surface characterization techniques (e.g., scanning electron microscopy, or coating thickness analysis) before and after exposure is recommended to confirm coating durability and its impact on antimicrobial efficacy.

## CONCLUSION

1. Orthodontic archwires coated with nanochitosan demonstrated significant antibacterial effects, reducing *S. mutans* counts by 1.70 × 10^6^ CFU/ml (95% CI: -1.8 to -1.5) and *L. acidophilus* counts by 1.60 × 10^6^ CFU/ml (95% CI: -1.84 to -1.36) compared with uncoated wires.
2. While these findings suggest a potential role in reducing bacterial factors associated with white spot lesion development, direct prevention of lesions was not assessed in this study. Future long-term investigations are required to evaluate this hypothesis and to further examine the antimicrobial efficacy, mechanical integrity, and bio-corrosion resistance of nanochitosan-coated orthodontic components.

## Data Availability

All data generated or analyzed during this study are included in this published article.
